# Neuromyelitis optica spectrum disorder after post appendectomy peritonitis: A case report

**DOI:** 10.1016/j.radcr.2024.09.078

**Published:** 2024-10-03

**Authors:** Muhammad Zaid, Fatima Saleemi, Rana Muhammad Usama, Ayman Tahir, Aima Azhar, Mohammed Mahmmoud Fadelallah Eljack, Muhammad Junaid Tahir

**Affiliations:** a Department of Internal Medicine, Lahore General Hospital, Lahore, Pakistan; b Department of Neurology, Punjab Institute of Neurosciences, Lahore, Pakistan; c Department of Internal Medicine, District Headquarters Hospital, Chakwal, Pakistan; d Department of Internal Medicine, Fatima Jinnah Medical University, Lahore, Pakistan; e Department of Internal Medicine, Faculty of Medicine and Health Sciences, University of Bakht Alruda, Eldueim, Sudan; f Department of radiology, Shaukat Khanum Memorial Cancer Hospital and Research Center, Lahore, Pakistan

**Keywords:** Magnetic resonance imaging, Spinal cord, Neurology, Antibodies, Plasmapheresis

## Abstract

Neuromyelitis optica spectrum disorder (NMOSD), also known as “Devic's syndrome”, is an autoimmune demyelination disorder. It affects the optic nerve and spinal cord, causing optic neuritis and transverse myelitis. It is associated with anti-aquaporin 4 antibodies that target the aquaporin channel on astrocytes. An 18-year-old male with a history of appendectomy 6 months ago presented with neck pain, numbness in limbs up to the umbilicus, paresis, and spasticity in the left leg. The brain magnetic resonance imaging (MRI) was normal, while the spinal cord MRI showed hyperintense foci at the T2 level. Cerebrospinal fluid (CSF) analysis was in the normal range and negative for oligoclonal bands. The serological assay was positive for anti-aquaporin-4 antibodies (AQP4-IgG). The patient improved significantly after administering high doses of methylprednisolone and supplements. Due to the unavailability of eculizumab, he underwent plasmapheresis sessions to remove antibodies, which improved to a reasonable extent**.** NMOSD most commonly targets older females, but in our report, it appeared in a young male. The patient only presented with transverse myelitis with no ophthalmologic problem. He made significant improvement with combination treatment of steroids, supplements, and plasmapheresis.

## Introduction

Neuromyelitis optica spectrum disorder (NMOSD), also named “Devic's syndrome”, is an autoimmune disorder characterized by optic neuritis, transverse myelitis, and brain stem lesions [1]. In most cases, it is a demyelination disorder caused by pathogenic serum IgG antibodies against aquaporin-4 (AQP4). AQP4-antibody differentiates neuromyelitis optica from multiple sclerosis. It binds to AQP4 channels on astrocytes, triggering activation of the classical complement cascade, causing granulocyte, eosinophil, and lymphocyte infiltration, culminating in injury first to astrocyte, then oligodendrocytes followed by demyelination and neuronal loss [2].

Disease manifestation can be monophasic or relapsing, affecting the optic nerve, spinal cord, area postrema, brainstem, diencephalon, and cerebellum [3]. Patient presents with optic neuritis, postrema syndrome (nausea, hiccups, and vomiting), myelitis (involving motor, sensory and sphincter control pathways), brain stem encephalitis, pain syndrome and cognitive dysfunctions [4].

Magnetic resonance imaging (MRI) findings indicative of NMOSD include extensive lesions along the optic nerve and spinal cord spanning 3 or more vertebral segments. Additionally, NMOSD can be suggested by bilateral optic nerve lesions, as well as lesions affecting the optic chiasm, area postrema, floor of the fourth ventricle, periaqueductal gray matter, hypothalamus, and walls of the third ventricle [5].

The possible symptoms of NMO includes pain in the eyes, loss of vision, weakness or numbness in the arms and legs, paralysis of the arms and legs, difficulty controlling the bladder or bowels, uncontrollable vomiting and hiccups [6]. Diagnosis of neuromyelitis optica involves MRI of the brain and spinal, cord and by testing blood serum for AQP4-antibodies. Physical and neurological examination is also essential because 13.5% of people do not have serum antibodies [7].

NMOSD is distributed worldwide, with an incidence that is higher in women than in men and more prevalent in Asian and African countries than in Western countries. Also, the peak incidence of disease ranges from 45 to 49 years of age [8].

## Case presentation

An 18-year-old male, presented to the outpatient department with progressive numbness in limbs up to umbilicus, weakness, and spasticity on left leg 6 months after suffering from peritonitis postappendectomy. His sphincter functions were completely normal. He had a history of neck pain for the last 2 months. There is no prior history of fever, loss of consciousness, seizures, and sensory loss. He denied any history of trauma or a previous similar episode. There was no significant past medical or surgical history. Family history was negative for any neurologic disorders.

**Fig. 1:**
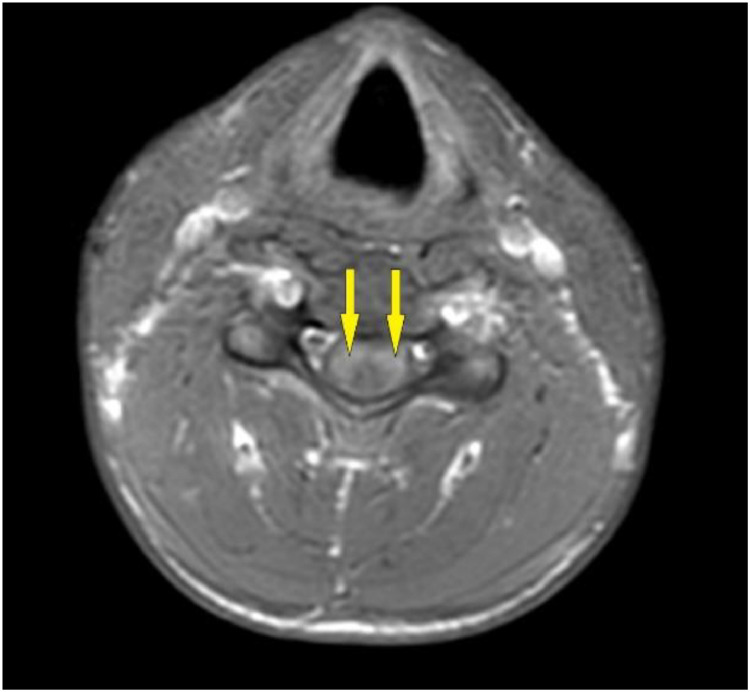
Axial MRI cervical spine T1 post contrast FAT SAT image showing enhanced peripherally placed lesion in upper cervical region.

**Fig. 2:**
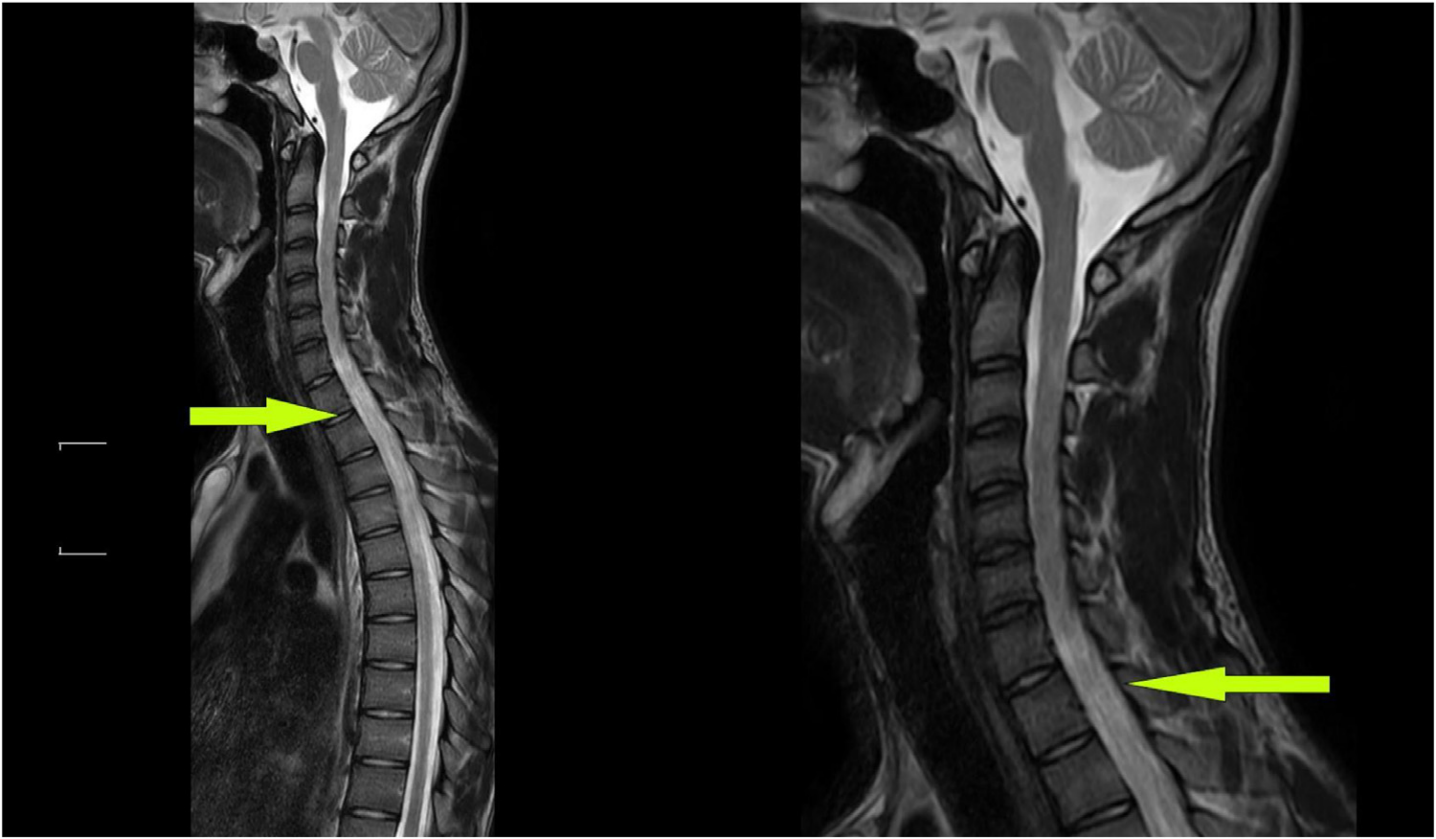
T2 sagittal FAT SAT MRI images showed intramedullary long segment hyperintense signals extending from C3 to T3 level of spinal cord.

Suspicion of lesions in the cervical and thoracic spine was made. Hence, MRI brain and cervical and thoracic spine with intravenous contrast was advised which showed T2 hyperintense lesions in the respective regions (C3 to T3 level of spinal cord) and a tentative diagnosis for transverse myelitis or ascending polyneuropathy was made until further investigations (Figs. 1 and 2). No brain parenchymal abnormality was seen. Drugs like methylprednisolone, muscle relaxants and supplements were administered to the patient promptly. To prevent neuropathy vitamin B12 was given adjunctively.

Because of the history of acute infection a few months ago, post infection myelitis was also suspected. Cerebrospinal fluid (CSF) analysis was normal, having clear consistency with proteins, red blood cells, white blood cells, and glucose in normal ranges. It was also negative for oligoclonal bands. Blood chemistry was completely normal. Blood, stool, and urine cultures for various infections were performed which came out negative for any sort of infection.

T2 hyperintense lesions were predictors for demyelination diseases like transverse myelitis, acute disseminated encephalomyelitis (ADEM), multiple sclerosis, neuromyelitis optica, intramedullary tumor and ascending polyneuropathy. To reach a diagnosis, an immunological assay was done which came out positive for anti-aquaporin-4 antibodies. This led to the confirmation of neuromyelitis optica which is a rare autoimmune demyelination disease. The patient underwent 5 sessions of plasmapheresis on alternative days for removal of AQP4 antibodies and was treated further by steroids, supplements, and muscle relaxants. With all this combination therapy his condition improved significantly over time.

## Discussion

NMOSD is an idiopathic demyelinating disease of the central nervous system that preferentially affects the optic nerve and spinal cord. Some significant symptoms are eye pain, loss of vision, weakness in the arms and legs, pain in the arms or legs – described as sharp, burning, shooting or numbing, increased sensitivity to cold and heat, tight and painful muscle spasms in the arms and legs, vomiting and loss of bladder and bowel control [9]. NMOSD is more common in women (>80 per cent) than men. It is more common among people of African and Eastern Asian descent than in people who are white. NMOSD, especially cases seropositive for AQP4-IgG, occur in late middle-aged women [10]. Seronegative cases have also been reported so far [11].

Many cases of onset or exacerbation of NMOSD have been described after infection with Mycobacterium tuberculosis, EBV and human immunodeficiency virus; however, no definite association with a virus has been found [12]. Similarly, in this case report, the patient underwent appendectomy and later had acute peritonitis a few months prior to the manifestation of symptoms of neuromyelitis optica.

The subject had blood and CSF analysis in completely normal range; oligoclonal bands were absent, but serology was positive for anti-aquaporin-4 antibody, which we suspect was an immune response triggered by peritonitis. This led to the discussion that viral infections and surgery can initiate an autoimmune response [13]. The most plausible mechanism that could explain the role of infections in triggering autoimmunity is molecular mimicry. Molecular mimicry occurs when proteins or other molecules from infectious agents or other external sources resemble self-proteins or antigens. When the immune system responds to the infectious agent, it may also inadvertently target self-proteins due to their similarity, leading to their presentation by antigen-presenting cells to autoreactive CD4+ T cells [14].

The subject was treated with high doses of intravenous steroids, muscle relaxants, supplements and plasmapheresis and improved significantly. The standard treatment for acute and chronic disease is different. During acute attacks, high-dose corticosteroids (most commonly intravenous methylprednisolone 1000 mg daily for 3-7 days), usually followed by tapered oral steroids, therapeutic plasma exchange (TPE) or immunoadsorption procedures, are also recommended. Food and Drug Administration (FDA) recently approved 3 drugs for the prevention of relapse, anti-C5 eculizumab, anti-IL- 6 satralizumab, and anti-CD19 inebilizumab [15].

NMOSD can be monophasic or relapsing. Predictors for prognosis are gender, age, history of disease and intervals of recurrent attacks. The prognosis is good in early diagnosis and monophasic disease conditions. Prognosis is poor when the disease relapses within the first 2 years after onset, older age at onset, female sex, and a history of other autoimmune diseases [16]. Patients who are diagnosed later or have relapsing NMOSD are also likely to have recurrent attacks of optic neuritis and myelitis. These lead to severe neurologic sequelae and impairment, including blindness and paraplegia.

## Conclusion

NMOSD is a complex neurological disorder primarily affecting women and certain ethnic groups. This case highlights potential triggers like infections and surgery, leading to autoimmune responses and highlights the importance of MRI for the diagnosis and better understanding of NMOSD. Prompt diagnosis and treatment improve outcomes, but prognosis varies based on age, gender, and disease history. Ongoing monitoring and treatment are crucial to prevent recurrent attacks and severe neurological complications. Recent FDA approved drugs might offer promising options for relapse prevention, indicating an evolving approach to managing NMOSD.

## Author contributions

MZ and RMU conceived and designed the case report, and were responsible for data collection and acquisition of data. FS, RMU, AT, AA, and MJT performed the literature review and wrote the manuscript. MJT reviewed and critically revised the manuscript. All authors have approved the final manuscript.

## Patient consent

Written informed consent was obtained from the patient's elder brother for publication and any accompanying images. A copy of the written consent is available for review by the Editor-in-Chief of this journal on request.
